# Cardiac metastasis in papillary thyroid carcinoma: a case report and literature review

**DOI:** 10.3389/fonc.2025.1590186

**Published:** 2025-05-13

**Authors:** Xiaonuo Zhang, Xinyu Huang

**Affiliations:** Department of Medical Oncology, Hangzhou Cancer Hospital, Affiliated Hangzhou First People's Hospital, West Lake University, Hangzhou, China

**Keywords:** papillary thyroid carcinoma (PTC), cardiac metastasis, clinical manifestation, prognosis, treatment

## Abstract

Metastasis of malignant tumors to the heart is rare in clinical practice, particularly in papillary thyroid carcinoma (PTC). This report presents the case of a male patient diagnosed with PTC who underwent radical surgery and received I131 treatment. During the fourth year of follow-up, he developed cervical lymph node metastasis. Despite systemic treatment, the patient was hospitalized in the seventh year due to a recurrent cough and expectoration. Anti-infective therapies proved ineffective, and imaging revealed lung metastasis. An echocardiogram identified a hypoechoic mass measuring 5.3 cm by 3.2 cm in the right ventricle, attached to the lateral wall and between the anterior and posterior leaflets, indicating cardiac metastasis. Following diagnosis, the patient’s condition deteriorated rapidly, culminating in death due to heart failure and severe infection. It is well-established that thyroid cancer can metastasize through both hematogenous and lymphatic pathways, with the lungs and brain being the most common sites, followed by the bones and liver. Cardiac metastasis, however, is exceedingly rare, and clinical reports are scarce. This case underscores the importance of considering atypical metastatic sites in advanced thyroid cancer and highlights the aggressive potential of the disease in certain patients. We hope this case raises awareness among oncologists.

## Introduction

Thyroid cancer is one of the most common endocrine malignancies, and its incidence has been rising steadily and rapidly in many countries and regions in recent years (1). This increase is primarily due to a rise in small, low-risk papillary thyroid cancers. Statistics indicate that there will be 44,280 new cases of thyroid cancer diagnosed in 2021, with nearly 85% of these cases classified as papillary thyroid carcinoma (2). The main pathological types include papillary carcinoma, follicular carcinoma, medullary carcinoma, and undifferentiated carcinoma (3). Among these, PTC is the most prevalent and is generally associated with a favorable prognosis, earning it the nickname “gentle cancer.” However, a subset of patients still experiences poor outcomes (4). Research indicates several factors are associated with Progression-Free Survival (PFS) time in thyroid cancer patients. These factors include the maximum tumor diameter, the number of cancer sites, lymph node metastasis, age at diagnosis, the recurrence of cancer, and distant metastasis. Notably, having more than five positive lymph nodes is a significant independent predictor of PFS (5).

The probability of distant metastasis in PTC is relatively low, with the most common sites of metastasis being the lungs, bones, and brain (6). Cardiac metastasis from PTC is exceptionally rare, and metastasis to the heart from other types of tumors is also uncommon in clinical practice. Metastatic tumors are more common than primary cardiac tumors among cardiac tumors (7). The most common primary cardiac tumor is the benign atrial myxoma. Among primary malignant cardiac tumors, sarcomas are more prevalent than carcinomas (8). Metastatic cardiac tumors most frequently originate from lung cancer (37%), followed by breast cancer (7%), esophageal cancer (6%), and hematologic malignancies, such as lymphoma (20%) (9). This variation is attributed to the high velocity of blood flow within the heart chambers, which makes it difficult for harmful substances, including cancer cells, to implant and establish metastatic lesions. Clinical manifestations of cardiac metastasis vary depending on the location and size of the tumor, and once it occurs, it significantly impacts the patient’s prognosis. Here, we report a patient of papillary thyroid carcinoma with multiple metastases, including cervical lymph node, lung, and cardiac metastases.

## Case reports

A 58-year-old man presented to the hospital following the discovery of a mass in his left neck. He reported no family history of cancer and no known drug allergies. On April 19, 2016, he underwent a modified radical resection for left thyroid cancer under general anesthesia. Postoperative pathological examination revealed papillary thyroid carcinoma (PTC) in the left lobe and isthmus of the thyroid, measuring 2 cm × 1.5 cm × 1.5 cm. The tumor had invaded the thyroid capsule, but no vascular invasion and metastatic involvement was identified in 5 out of 22 lymph nodes examined from the left neck. Immunohistochemical analysis showed the following markers: CK (+), TTF-1 (+), TG (+), Galectin-3 (+), HBME-1 (+), TPO (-), HCK (+), and P53 (-). Based on these findings, the postoperative pathological staging indicated pT2N1bM0, corresponding to stage II. The patient received iodine-131 radiotherapy for five days and was placed on Euthyrox replacement therapy. Regular follow-up examinations showed that the patient’s condition remained stable. However, in 2020, the patient accidentally discovered a mass on the left side of his neck. A needle biopsy of the left cervical lymph node confirmed the presence of metastatic papillary thyroid carcinoma.

**Figure 1:**
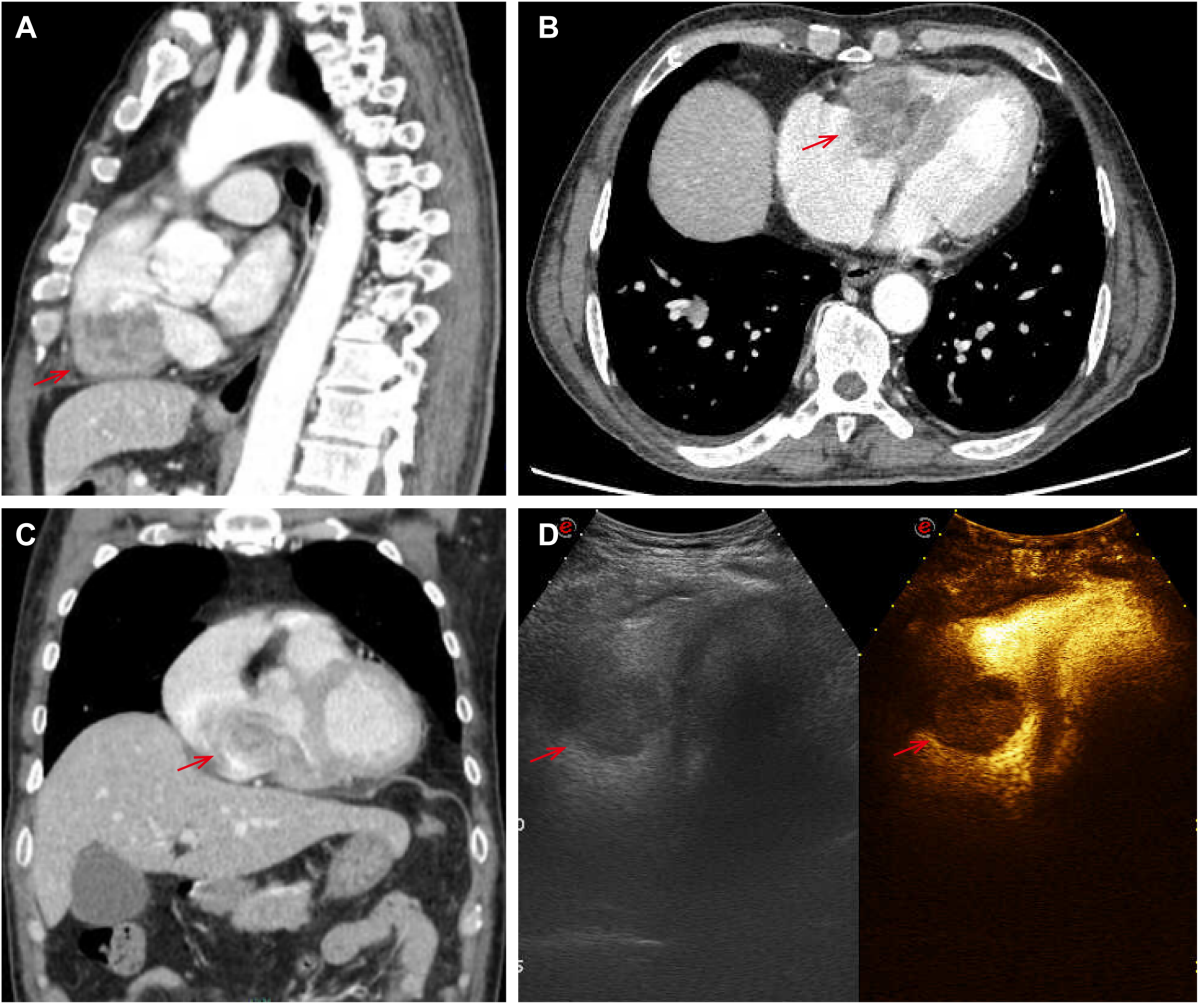
Pictures **(A–C)** showed cardiac metastasis on the right ventricle at chest-enhanced CT scans; the echocardiogram showed a hypoechoic mass of 5.3*3.2cm in the right ventricle on picture D.

**Figure 2:**
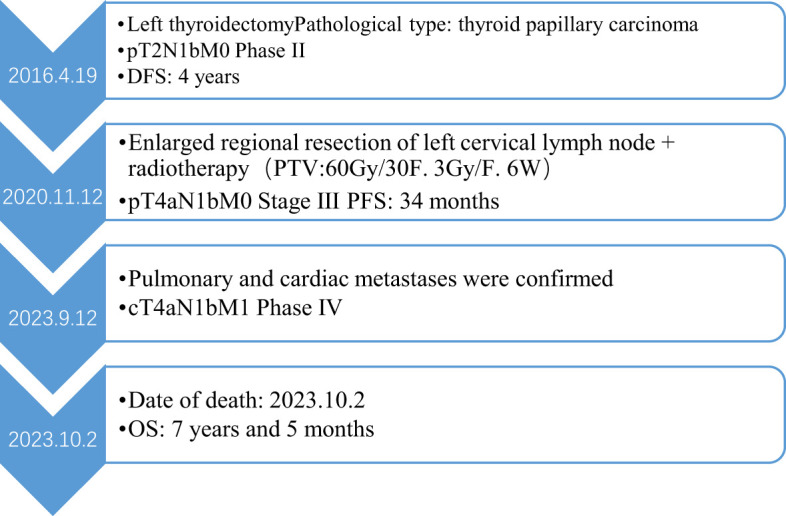
The case timeline.

There was no doubt that the tumor had metastasized to the left side of the neck, prompting the decision to perform an extended regional lymph node excision. Postoperative pathological staging revealed pT4aN1bM0, classified as stage III. Following surgery, the patient underwent radiotherapy for the cervical lymph nodes beginning on November 30, 2020. The treatment plan targeted the left neck regions II and IV, delivering a total dose of 60 Gy over 30 fractions at a rate of 3 Gy per fraction for 6 weeks. However, since August 2023, the patient began experiencing a persistent cough and expectoration without an apparent cause, which did not respond to antibiotic treatment. Additionally, he developed bilateral lower limb edema, chest tightness, and shortness of breath during physical activity. A chest CT scan revealed multiple metastases in both lungs. An echocardiogram performed on September 10, 2023, showed a hypoechoic mass measuring 5.3 × 3.2 cm in the right ventricle. The mass was attached to the lateral wall of the right ventricle, situated between the anterior and posterior leaflets. The electrocardiogram indicated sinus tachycardia with a heart rate of 102 beats per minute. Laboratory results showed significantly elevated thyroglobulin (TG) levels (>500 ng/ml) and increased tumor markers: NSE at 31.59 ng/ml, CA-125 at 124.5 U/ml, and CA 19–9 at 138.97 U/ml, all exceeding the upper limits of normal. Cardiac metastasis was further confirmed through contrast-enhanced echocardiography and chest-enhanced CT scans. (**Figure 1**). The patient’s condition deteriorated rapidly, with symptoms of cardiac insufficiency, including lower limb edema, chest tightness, shortness of breath, and orthopnea, as well as coagulopathy secondary to a severe infection. Despite efforts to manage these complications, the patient passed away one month after the discovery of cardiac metastasis. A timeline of the case is presented in **Figure 2**.

## Discussion

Papillary thyroid carcinoma (PTC) is a common malignancy and typically carries a favorable prognosis, with studies reporting a 10-year survival rate ranging from 98.1% to 98.7% (5). However, survival outcomes can vary significantly depending on several prognostic factors. Research indicates that tumor size, the number of lymph node metastases, recurrent disease, and distant metastases are critical factors influencing PFS. Furthermore, distant metastases, recurrent cancer, and patient age at diagnosis have been shown to impact overall survival (OS) (10). A retrospective study on the prognostic significance of lymph node metastasis in PTC reveals that even a single small lymph node metastasis can elevate the risk of recurrence in low-risk patients with papillary thyroid microcarcinoma (11). The study found that the recurrence rate was 2% in patients with no initial clinical lymph node metastasis (N0), compared to 22% in patients with initial clinical lymph node metastasis (N1). Among patients classified as pathological N1, the recurrence rate was 4% for those with fewer than five positive lymph nodes. Still, it rose significantly to 19% for patients with more than five positive lymph nodes (12).

In this case, the patient was diagnosed with PTC at over 55 years of age, with a maximum tumor diameter of 2 cm. The cancer had extended beyond the thyroid capsule, and the patient had more than five positive lymph nodes, all of which are recognized as high-risk factors for recurrence and poor prognosis. Four years after undergoing radical surgical resection, the patient developed cervical lymph node metastasis, and by the seventh year post-diagnosis, lung and cardiac metastases were detected. This progression highlights that while radical surgical resection is a cornerstone of treatment for PTC, it does not guarantee a permanent cure, particularly in patients with high-risk features. The emergence of distant metastasis, including the rare occurrence of cardiac involvement, underscores the aggressive potential of PTC in certain cases and the need for long-term surveillance and multidisciplinary management.

Cardiac metastasis from thyroid cancer most commonly occurs in the right ventricle, although other sites such as the right atrium, pericardium, left atrium, and left ventricle can also be involved. There are three primary mechanisms by which tumors spread to the heart: hematogenous spread, lymphatic spread, and direct extension, as well as intracavitary spread through the superior and inferior vena cava (13). Hematogenous spread typically affects the myocardium, while lymphatic spread is more likely to involve the pericardium. Tumors can invade the entire heart, with both the epicardium and myocardium being commonly affected. Notably, the right side of the heart is more frequently impacted than the left, likely due to its anatomical and hemodynamic characteristics. In this case, the findings are consistent with right ventricular metastasis, as evidenced by the hypoechoic mass measuring 5.3 × 3.2 cm in the right ventricle. Given the patient’s lung metastasis, it is likely that the right ventricular metastasis resulted from the tumor spreading from the primary site through blood or lymphatic pathways. Continuous diffusion from other metastatic sites is the most plausible explanation for this cardiac involvement.

The clinical manifestations of cardiac tumors vary widely and depend on several factors, including tumor size, anatomical location, involvement of heart valves, hemodynamic impact, and the presence of pericardial metastasis (13). Asymptomatic patients may accidentally discover these tumors during imaging studies. Symptomatic patients may present with different symptoms, which depend on the tumor’s location within the heart. Tumors located in the right atrium and right ventricle can obstruct blood flow, leading to symptoms such as venous congestion, and pulmonary embolism. The latter occurs when tumor fragments or thrombi dislodge and travel to the pulmonary circulation. In contrast, tumors in the left atrium or left ventricle are more likely to cause systemic embolization, such as cerebral infarction or peripheral arterial occlusion, due to the dissemination of tumor fragments or thrombi into the systemic circulation. Additionally, pericardial metastasis can result in increased pericardial effusion, potentially leading to complications like cardiac tamponade and arrhythmias.

Echocardiography is the primary diagnostic method used for suspected cardiac masses. Computed tomography (CT) provides high-resolution cross-sectional imaging, which allows for the clear identification of these masses (8). In patients with a history of thyroid cancer who do not exhibit cardiac symptoms, any new changes in an electrocardiogram (ECG) excluding underlying diseases and abnormal hormone levels, should trigger concerns about potential cardiac metastasis. An example from the literature describes a 70-year-old woman who developed a new case of atrial fibrillation; the final diagnosis was metastatic thyroid cancer to the heart (14).

The main treatment options for cardiac metastasis include chemotherapy, surgical intervention, and brachytherapy. However, survival rates after diagnosing cardiac metastasis are generally minimal. A large-scale review of cardiac metastasis stemming from cervical cancer, which included 86 cases, confirmed that the heart is a rare site for metastasis. Once cardiac metastasis is diagnosed, the prognosis is extremely poor, with a median survival of only three months (15). Therefore, aggressive treatment may be questionable, and personalized decisions should be made based on each patient’s specific circumstances. Palliative care and symptom management often play a central role in improving the quality of life for these patients.

This case serves as a reminder that even in cancers with generally favorable outcomes, high-risk patients require close monitoring and tailored therapeutic strategies to address the possibility of recurrence and metastasis. By maintaining a high index of suspicion in cancer patients, particularly those with high-risk features or advanced disease, clinicians can identify cardiac involvement at an earlier stage, enabling timely intervention and personalized treatment strategies.

## Data Availability

The original contributions presented in the study are included in the article/supplementary material. Further inquiries can be directed to the corresponding author.
