# Correction: Cardiac metastasis in papillary thyroid carcinoma: a case report and literature review

**DOI:** 10.3389/fonc.2025.1634281

**Published:** 2025-06-25

**Authors:** Xiaonuo Zhang, Xinyu Huang

**Affiliations:** Department of Medical Oncology, Hangzhou Cancer Hospital, Affiliated Hangzhou First People’s Hospital, West Lake University, Hangzhou, China

**Keywords:** papillary thyroid carcinoma (PTC), cardiac metastasis, clinical manifestation, prognosis, treatment

In the published article, the author list was only “Xiaonuo Zhang”. The correct author list is “Xiaonuo Zhang, Xinyu Huang”.

In the published article, the corresponding author’s name incorrect is “Xiaonuo Zhang”. The correct name is “Xinyu Huang”.

In the published article, there was an error in the author list, and author “Xinyu Huang” was erroneously excluded. The corrected author list appears below.

“Xiaonuo Zhang, Xinyu Huang

Xiaonuo Zhang completed case screening, data collection, literature review, and wrote the manuscript.

Xinyu Huang supervised the project and revised the manuscript.”

The authors apologize for this error and state that this does not change the scientific conclusions of the article in any way. The original article has been updated.

